# A Citrus Peel Waste Biorefinery for Ethanol and Methane Production

**DOI:** 10.3390/molecules24132451

**Published:** 2019-07-04

**Authors:** Maria Patsalou, Charis G. Samanides, Eleni Protopapa, Stella Stavrinou, Ioannis Vyrides, Michalis Koutinas

**Affiliations:** Department of Environmental Science & Technology, Cyprus University of Technology, 30 Archbishop Kyprianou Str., 3036 Limassol, Cyprus

**Keywords:** bioethanol, biomethane, citrus peel waste, biorefinery, biorefinery residues

## Abstract

This paper deals with the development of a citrus peel waste (CPW) biorefinery that employs low environmental impact technologies for production of ethanol and methane. Three major yeasts were compared for ethanol production in batch fermentations using CPW pretreated through acid hydrolysis and a combination of acid and enzyme hydrolysis. The most efficient conditions for production of CPW-based hydrolyzates included processing at 116 °C for 10 min. *Pichia kudriavzevii* KVMP10 achieved the highest ethanol production that reached 30.7 g L^−1^ in fermentations conducted at elevated temperatures (42 °C). A zero-waste biorefinery was introduced by using solid biorefinery residues in repeated batch anaerobic digestion fermentations achieving methane formation of 342 mL g_VS_^−1^ (volatile solids). Methane production applying untreated and dried CPW reached a similar level (339–356 mL g_VS_^−1^) to the use of the side stream, demonstrating that the developed bioprocess constitutes an advanced alternative to energy intensive methods for biofuel production.

## 1. Introduction

Vegetable and fruit waste account for 20–50% of household waste in various countries, while citrus peel waste (CPW) comprises a principal residue under the specific category [1]. Global citrus production (orange, lemon, lime, kinnow, sweet orange, etc.) constitutes over 121 × 106 t annually, while the industrial juice manufacturing sector generates about 25 × 10^6^ t of CPW [2]. CPW formed during processing of the fruit consists mainly of peels and pressed pulp (seeds and segment membranes), accounting for 50% of the fruit’s weight [3,4]. 

Current management options for CPW include burying in landfills and application as animal feed after drying. However, the thermal dehydration process of CPW to produce animal feeds is energy consuming and not always cost-effective [5], while the final product consists of rather poor animal feed, due to low protein content and high quantity of sugars [6]. Moreover, the waste includes elevated organic matter (approximately 95% of total solids) and water content (approximately 80–90%), as well as low pH (3–4), making CPW inappropriate for landfilling based on the latest EU Waste Framework Directive 2008/98/EC [7]. 

Although the disposal of CPW is opposed to EU regulations, the waste could serve as a valuable feedstock for the manufacture of biofuels and other commodities. Specifically, CPW comprises high levels of pectin, cellulose, hemicellulose, and soluble sugars, while 0.5% g g^−1^ of essential oils are included on a wet basis [8,9]. D-limonene, constituting the main component of essential oils in CPW, is a terpenic compound with antimicrobial properties and applicability in food, cosmetics, and pharmaceutical industries [10]. Thus, citrus processing industries usually recover essential oils from the waste streams produced as an added-value extractable component [11].

Global climate change associated with the extensive release of greenhouse gases has raised concerns about the application of fossilized hydrocarbons as the main energy source [12]. Thus, in recent years, exploitation of new renewable resources for the production of biofuels as a replacement for the use of the nonrenewable source of petroleum has received much global research interest [13]. The ability of microorganisms to use renewable resources for biofuel synthesis is exploited by the current industry manufacturing biofuels (e.g., bioethanol, biomethane, biobutanol), mainly from sugarcane, corn, and wheat [14]. However, various studies have investigated the application of CPW as a promising feedstock for biofuel production through bioprocessing, using different pretreatment approaches and microorganisms.

Due to the antimicrobial properties of d-limonene, CPW valorization for production of ethanol and methane requires the removal of essential oils to avoid biosystem inhibition [10,15]. Various pretreatment methods have been employed for extraction of essential oils, including steam explosion [10,16,17], hydrothermal sterilization [15,18], popping [19], and drying [20,21]. However, essential oils and pectin constitute high added-value commodities, which were not extracted in several existing studies. Other CPW-based valorization approaches produced extractables and fermentation products using energy intensive approaches, including elevated acid hydrolysis temperatures, low ethanol fermentation temperatures that contribute to additional cooling and separation costs, and anaerobic digestion under thermophilic conditions.

The current study explored alternative and low environmental impact technologies for CPW valorization through the development of a biorefinery strategy. Low temperature dilute acid hydrolysis as a single procedure and combined with enzyme hydrolysis was evaluated as a CPW pretreatment method, following extraction of essential oils (via distillation) and pectin. The hydrolyzate formed was tested for ethanol production employing the thermotolerant strain *Pichia kudriavzevii* KVMP10, which was compared with two industrial yeasts at elevated fermentation temperatures, aiming to reduce biofuel production costs. A zero-waste process was targeted, employing solid biorefinery residues (BR) for methane production, which was compared with the use of CPW and dried citrus peel waste (DCPW), constituting the first study to our knowledge comparing the specific citrus waste-based fractions in anaerobic digestion.

## 2. Results and Discussion

### 2.1. The CPW Biorefinery Strategy

**Figure 1:**
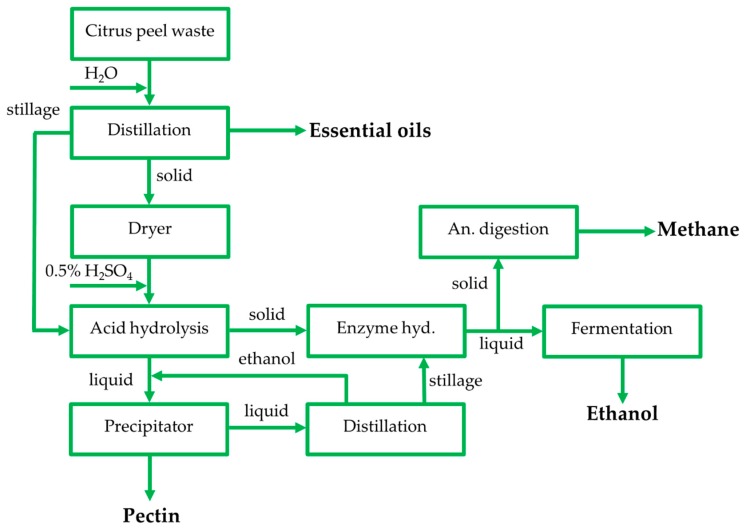
Process flow sheet of the zero-waste biorefinery used for citrus peel waste (CPW) valorization.

The current biorefinery aimed for CPW valorization through the removal of essential oils and pectin, as well as fermentation of the hydrolyzate formed as fermentation feedstock for bioethanol production. A zero-waste concept was established by refinement of remaining solid fractions via anaerobic digestion for methane production. Specifically, the first step included extraction of essential oils using distillation, while the remaining solid residues of the process were dried and applied to dilute acid hydrolysis under varying conditions. Thus, pectin was extracted from the hydrolyzate generated through precipitation with the addition of ethanol, while the hydrolyzate was subsequently applied to distillation for ethanol removal. Following pectin isolation, the hydrolyzate was fermented to bioethanol, testing the effectiveness of three yeast strains (*P. kudriavzevii* KVMP10, *Kluyveromyces marxianus*, and *Saccharomyces cerevisiae*), while the remaining solid residues from dilute acid hydrolysis were anaerobically digested for methane generation (Figure 1).

### 2.2. Ethanol Production

The industrial production of ethanol is currently performed by catalytic hydration of ethylene (chemical method) [22] and by fermenting agricultural feedstocks (biochemical method) [14]. Ethanol can serve as a green energy source, which is mainly produced using starch, sugar, and carbohydrates, such as corn, potato, molasses, sugarcane, and lignocellulosic biomass [23]. Sugars can be directly converted to ethanol, while starchy and cellulosic materials should be first pretreated mainly using enzymes or chemicals to hydrolyze the polymers into sugars [24]. Herein, CPW has been applied as a cellulosic material, which includes an additional significant content of soluble sugars that could be applied for production of a hydrolyzate rich in carbon sources for ethanol fermentations. The thermotolerant strain *P. kudriavzevii* KVMP10 was tested and compared with two industrial yeasts (*K. marxianus* and *S. cerevisiae*) for ethanol production from CPW hydrolyzates under technologically favourable fermentation conditions. Thus, an elevated bioprocess temperature (42 °C) was applied in an attempt to reduce operational costs associated with decreased energy use for cooling and lower contamination risk [25,26].

#### 2.2.1. Ethanol Production Using CPW Hydrolyzates Obtained through Acid Hydrolysis 

**Figure 2:**
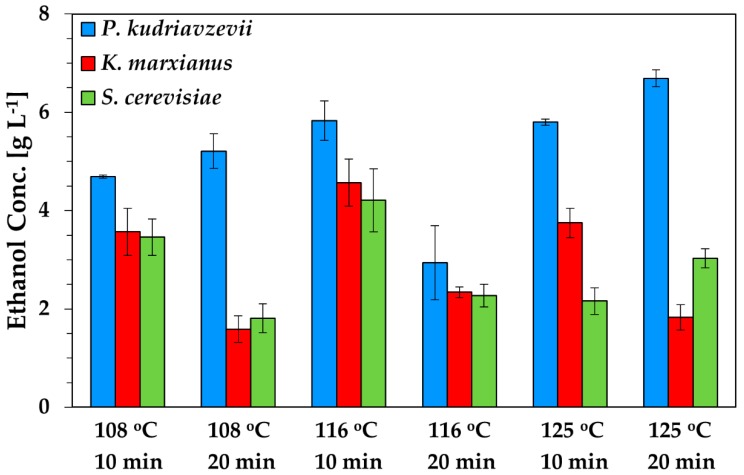
Εthanol titre achieved in *Pichia kudriavzevii* KVMP10, *Saccharomyces cerevisiae*, and *Kluyveromyces marxianus* fermentations of hydrolyzates obtained through dilute acid treatment of CPW.

Dilute acid hydrolysis of CPW served the dual objective of breaking down polymers (cellulose, hemicellulose) into soluble sugars, while consisting of an essential processing step for pectin isolation [27]. Thus, six CPW hydrolyzates were produced using three hydrolysis temperatures (108 °C, 116 °C, 125 °C) for 10 min and 20 min, respectively. Optimal conditions for CPW saccharification through dilute acid hydrolysis were identified as 116 °C for 10 min, based on the concentration of ethanol produced and the final product yield during fermentations (Figure 2, Table 1). Thus, ethanol concentration and product yield reached 5.8 g L^−1^ and 0.48 g_ethanol (eth)_ g^−1^_total sugar consumed (TSC)_, respectively, using *P. kudriavzevii* KVMP10, while *K. marxianus* and *S. cerevisiae* produced 4.6 g L^−1^ and 4.2 g L^−1^ of ethanol, respectively. Although, the highest ethanol concentration (6.7 g L^−1^) was obtained using the hydrolyzate generated at 125 °C for 20 min with application of *P. kudriavzevii* KVMP10, the product yield decreased significantly to 0.32 g_eth_ g^−1^_TSC_. A *t*-test (*p* < 0.05) was performed to statistically assess differences between the mean values of ethanol concentration. Significant statistical difference was observed between ethanol titres obtained in *P. kudriavzevii* KVMP10 and *S. cerevisiae* fermentations, using both hydrolyzates exhibiting the highest product formation (116 °C for 10 min and 125 °C for 20 min). Nevertheless, the titre of the biofuel obtained in *P. kudriavzevii* KVMP10 fermentations fed with the aforementioned hydrolyzates was not statistically different. Overall, in nearly all experiments performed using *K. marxianus* and *S. cerevisiae*, the increase in hydrolysis duration reduced the final product titre and yield, indicating the potential formation of inhibitors at elevated preprocessing duration. This is in line with previous studies demonstrating the inhibitory effect that may occur in ethanol fermentations of *K. marxianus* [28] and *S. cerevisiae* [29] fed with feedstocks pretreated employing increased acid hydrolysis temperatures and duration. Specifically, hydrolyzates derived from acid pretreatment resulted in low product yield in *K. marxianus* fermentations [28], while the presence of acid hydrolysis products (furan derivatives, weak acids, phenolics) inhibited the growth of *S. cerevisiae* and caused a reduction in ethanol yield and productivity [29]. Therefore, considering that the overall performance of *K. marxianus* and *S. cerevisiae* was decreased, the lower product yield of *P. kudrivzevii* KVMP10 and the elevated energy demand expected with application of 125 °C for 20 min in hydrolysis, the use of 116 °C for 10 min was selected as suitable conditions for CPW hydrolysis. These results are in agreement with other studies demonstrating that the optimal conditions for dilute acid hydrolysis of CPW comprise application of 116 °C for 10–13 min with the use of 0.5% (*v*/*v*) H_2_SO_4_ and 5–6% (*w*/*v*) dry solids of the raw material (rm) [30,31].

A variety of CPW pretreatment approaches have been previously evaluated for ethanol production by different yeasts. The production of the biofuel was investigated in *S. cerevisiae* fermentations, using a CPW-derived hydrolyzate obtained through application of steam explosion as well as dilute-acid hydrolysis and pectin recovery, achieving a yield of 0.43 g_eth_ g^−1^_TSC_ [16,32]. *S. cerevisiae* was also applied for bioethanol production using CPW hydrolyzates obtained through hydrothermal sterilization, steam explosion, and a combination of popping and enzyme hydrolysis, demonstrating ethanol production of 42 g L^−1^ [15], 60 L t^−1^_raw material_ [33] and 46.2 g L^−1^, respectively [19]. Orange peel waste was pretreated by two-stage acid and enzyme hydrolysis, while the hydrolyzate formed was used as fermentation feedstock for bioethanol production by *S. cerevisiae* and *Mucor indicus*, achieving final product concentrations of 30.3 g L^−1^ and 15 g L^−1^ with a yield of 0.46 g_eth_ g^−1^_TSC_ and 0.39 g_eth_ g^−1^_TSC_, respectively [21,34]. 

#### 2.2.2. Optimization of the Fermentation Process

The final ethanol titre achieved using the hydrolyzates obtained was low compared to the relevant literature. Therefore, a number of parameters were evaluated to improve ethanol production through the biorefinery proposed. The effect of nitrogen source supplementation was initially tested to increase biofuel production. However, although nitrogenous compounds can substantially enhance the fermentation rate of ethanol [35,36], the use of yeast extract as a nitrogen source did not demonstrate any noticeable effect on ethanol formation in all yeast fermentations conducted. 

Acid and enzyme hydrolysis were combined as sequential pretreatments to enhance the production of ethanol, due to potential increased release of fermentable sugars. The addition of the enzymatic pretreatment step in the biorefinery substantially enhanced the titre of bioethanol to 9.2 g L^−1^ in *P. kudriavzevii* KVMP10 fermentations, resulting in a product yield of 0.42 g_eth_ g^−1^_TSC_. Pretreatment processes that involve two steps can be more efficient in lignocellulosic biomass saccharification, releasing increased concentrations of simple sugars that enhance ethanol generation [19,21]. Thus, sequentially applied acid and enzyme hydrolysis of CPW was capable of producing substantially elevated sugar yields that reached 0.58 g_total sugar_ g^−1^_dry raw material_ [30]. 

Recycling of the remaining liquid stillage following essential oil extraction into the hydrolysis process was applied in an attempt to further increase the concentration of monosaccharides in the hydrolyzate and ethanol formation, as well as to reuse the process water generated for plant application. Stillage water recycling contributed to a three-fold increase of ethanol concentration that reached 30.7 g L^−1^ in *P. kudriavzevii* KVMP10 fermentations conducted at 42 °C, while biofuel production was also enhanced in *K. marxianus* cultures reaching 26.3 g L^−1^. Nevertheless, ethanol production from *S. cerevisiae* remained at low levels with the reuse of the stillage, potentially due to the elevated process temperature employed. The present findings demonstrate that *P. kudriavzevii* KVMP10 constitutes a more efficient ethanol producer with application of CPW hydrolyzates as compared to the industrial yeasts tested (*K. marxianus* and *S. cerevisiae*). Moreover, the increased ethanol production was achieved at an elevated fermentation temperature, which is in agreement with other studies highlighting that *P. kudriavzevii* strains are robust ethanol producers exhibiting multiple tolerance at high temperatures and acidic environments [37]. Key novelties of the results obtained are that high ethanol production was achieved from CPW under mild pretreatment and harsh fermentation conditions, while saving of valuable resources was achieved through process water recycling. Thus, in accordance with the water reuse achieved, the remaining solid residue from CPW hydrolysis was applied in anaerobic digestion to produce biogas targeting the operation of a zero-waste biorefinery as described below.

### 2.3. Anaerobic Digestion of Biorefinery Residues

The biomethanization of CPW has been mainly studied following essential oil extraction, which serves the dual purpose of generating an added-value product and reducing the anaerobic digestion inhibition caused by the antimicrobial properties of the oil [5]. However, although essential oils were extracted from CPW in several studies prior to use in methane production [10,32], untreated CPW has also been used in anaerobic digestion of fresh or dried citrus fragments (peel, pulp, and seeds) under thermophilic [17,38] and mesophilic conditions [20,39]. Herein, methane production was tested using biorefinery solids BR, remaining as a side stream from acid hydrolysis of CPW, and their capacity to produce the biofuel was compared to the application of untreated CPW as well as DCPW under mesophilic conditions.

#### 2.3.1. Methane Production Using BR, CPW, and DCPW

**Figure 3:**
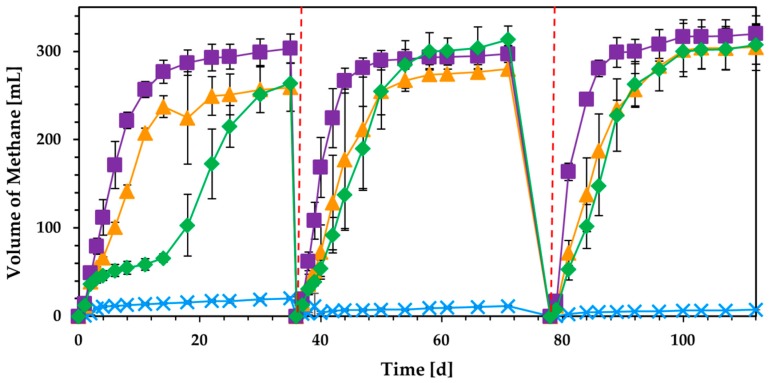
Cumulative methane production using 6 g_VS_ L^−1^ of untreated CPW (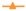), dried citrus peel waste (DCPW) (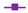), and solid biorefinery residues (BR) (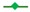). A control fermentation was conducted without the addition of CPW (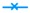), while all experiments were conducted at 37 °C. Dashed lines represent the time where substrate refeed was applied.

**Figure 4:**
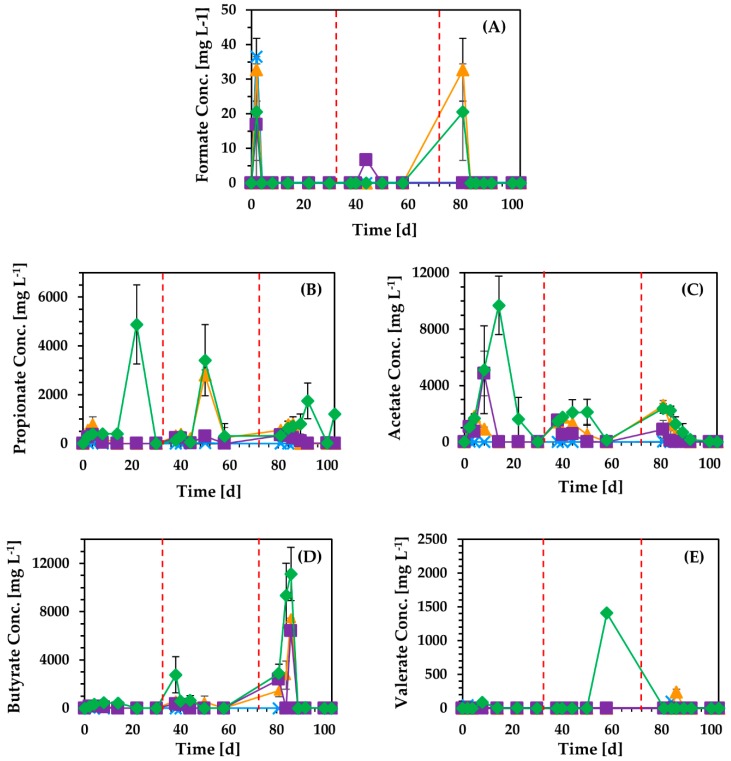
Concentration of volatile fatty acids, (**A**) formate, (**B**) propionate, (**C**) acetate, (**D**) butyrate, and (**E**) valerate, formed during anaerobic digestion of untreated CPW (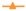), DCPW (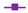), solid BR (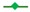), and in the control experiment (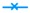).

Biogas and methane production from BR as well as CPW and DCPW was evaluated with the addition of 6 g L^−1^ as initial content of volatile solids (VS) in anaerobic digestion (Figure 3). During the first batch, DCPW exhibited substantially higher production of methane at 35 d, as compared to the rest of the materials, generating 303 mL. The use of BR demonstrated prolonged biogas inhibition for the first 15 days of the process, accumulating high levels of acetate that reached 9.7 g L^−1^ (Figure 4), while CPW also caused inhibition of methane formation. However, the concentration of acetate was subsequently reduced in the experiment fed with BR, resulting in methane production that reached 264 mL at 35 days, similarly to biofuel production from CPW. Following 36 days, a subsequent batch was conducted through refeeding of the same content of each material in fermentations. The inhibitory effect was substantially reduced in the second batch, regarding the kinetics of methane production, indicating potential adaptation of the sludge to each material. Specifically, the production rate of methane was 11.3 mL d^−1^, 15.9 mL d^−1^, and 7.5 mL d^−1^ during the first batch experiment, while in the second batch, the rate increased to 14.9 mL d^−1^, 25.6 mL d^−1^, and 13.6 mL d^−1^ for CPW, DCPW, and BR, respectively. Following the second refeed of each material, the methane production rate was further increased only in the digestion of DCPW, reaching 27.2 mL d^−1^. Moreover, cumulative methane formation was significantly increased with the use of BR, reaching 314 mL. Following 78 days, a second refeed of each material was applied to evaluate whether methane production was further increased. The production of methane was only slightly enhanced in the third batch, reaching 305–320 mL, for all materials tested. The data obtained demonstrate the capacity of BR to generate significant amounts of methane at a similar level to the use of dried or untreated CPW. Moreover, although methane production is consistent with other studies that reached similar biofuel formation from CPW [10,20,39], the production of the biofuel achieved here under mesophilic conditions was comparable to that of studies employing thermophilic anaerobic digestion temperatures [10,32]. Therefore, the approach followed not only enables the development of a zero-waste biorefinery, but also provides significant energy gains, due to the substantially reduced temperature used in anaerobic digestion. 

**Figure 5:**
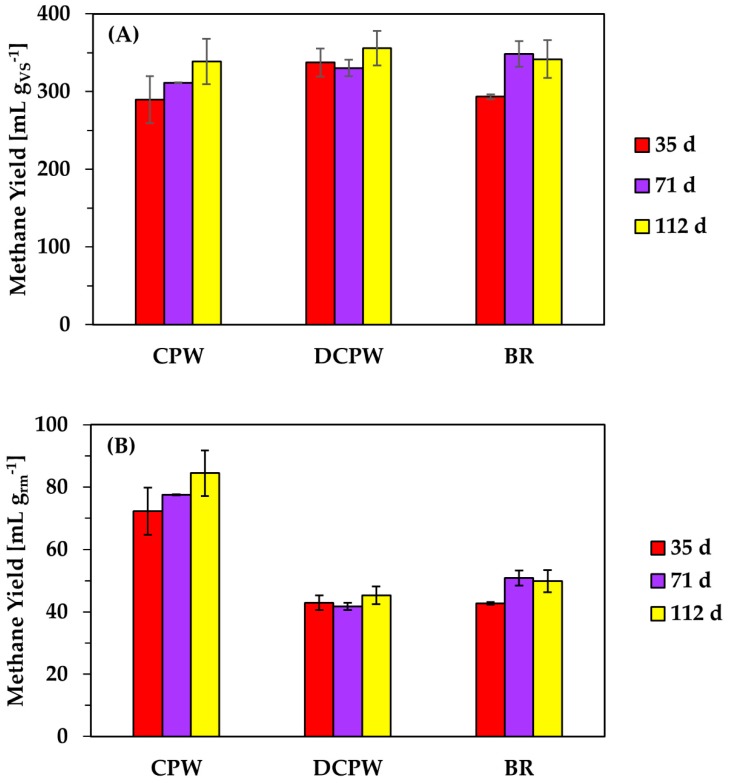
Yield of methane (**A**) as mL of product per g of initial volatile solids (VS) and (**B**) as mL of product per g of raw material (rm) generated from anaerobic digestion of CPW, DCPW, and BR following completion of each batch.

Although the use of DCPW in anaerobic digestion resulted in the highest production of methane in the first batch as compared to the application of CPW and BR, calculation of bioprocess yield showed that employing CPW in the system enhanced the overall waste-to-energy conversion (Figure 5). The methane yield, defined as mL of methane produced per g of raw material (rm) used, was substantially higher, with the application of untreated CPW ranging between 72 and 84 mL_methane_ g^−1^_rm_, respectively. However, employing DCPW and BR resulted in a similar and lower process yield that reached 42–51 mL_methane_ g^−1^_rm_ for both materials. The enhanced methane yield performed with the use of CPW was expected, given the higher organic content remaining in the specific material for treatment in anaerobic digestion as compared to DCPW and BR, where volatile compounds and/or hydrolyzed carbohydrates have been removed. This conclusion was clarified by the *t*-test (*p* < 0.05) performed to identify statistically significant differences in the mean values obtained for the final methane production (mL) per g of rm with each feedstock in the first two batches. Furthermore, a statistically significant increase (*p* < 0.05) was observed in the volume of biogas produced per g of rm between 35 days and 112 days with the use of BR, demonstrating the potential adaptation of the culture to the specific material following the first batch. Direct comparison of the three different citrus waste fractions demonstrated that even BR could produce high contents of methane, highlighting the applicability of biorefinery side streams for valorization through anaerobic digestion. 

#### 2.3.2. Biomethane Production Using Different Initial Quantities of CPW

**Figure 6:**
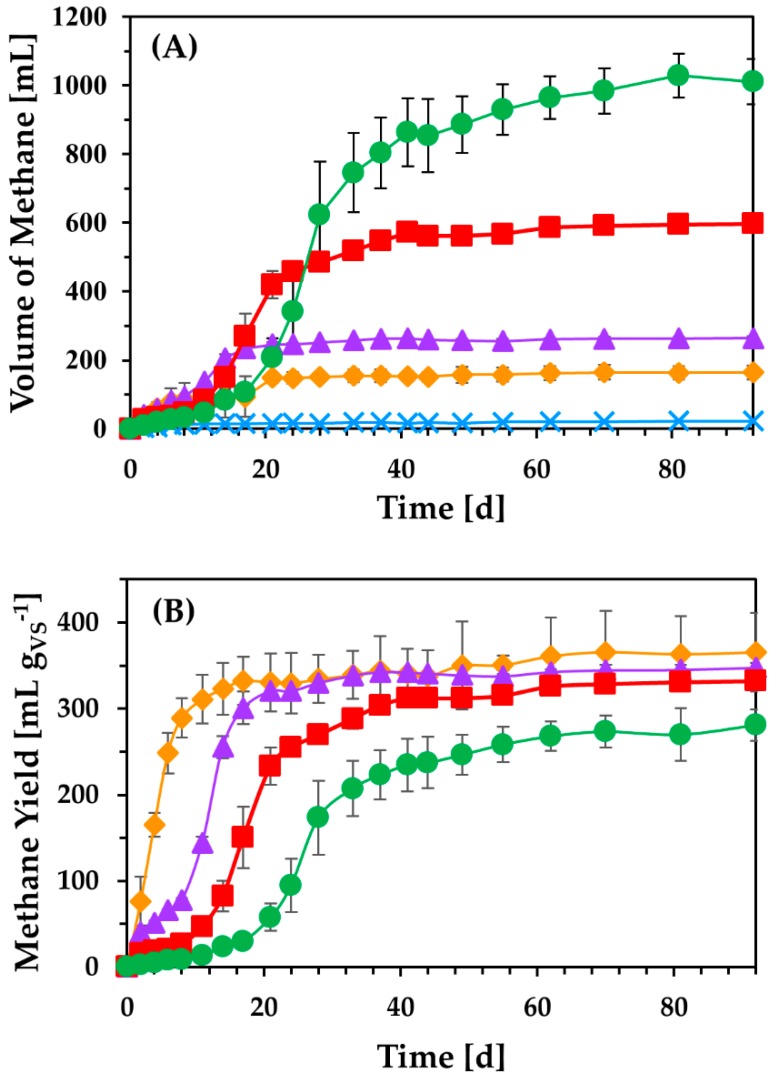
Cumulative methane production (**A**) and methane content per g of initial VS (**B**) using 3 g L^−1^ (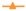), 6 g L^−1^ (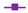), 12 g L^−1^ (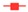), and 24 g L^−1^ (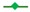) initial volatile solids of CPW. A control fermentation was performed without the addition of CPW (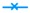), while all experiments were conducted at 37 °C.

Biomethanization of untreated CPW was previously evaluated by various studies. Forgacs et al. [17] achieved significantly low production of methane from untreated citrus waste that reached 102 mL g^−1^_VS_, while Koppar and Pullammanappallil [38] generated 644 mL g^−1^_VS_ from untreated CPW under thermophilic conditions. The use of peel, seeds, and pulp of fresh ripe orange as distinct feedstocks in anaerobic digestion under mesophilic conditions resulted in methane production that reached 72 mL g^−1^_VS_, 581 mL g^−1^_VS_, and 288 mL g^−1^_VS_, respectively [39]. Herein, different initial quantities of untreated CPW volatile solids (ranging between 3–24 g L^−1^) were applied in anaerobic digestion to evaluate the effect of increasing contents of organic material as well as essential oils in the bioprocess (Figure 6). The maximum cumulative production of methane in each experiment was monitored as 165 mL, 264 mL, 598 mL, and 1011 mL, employing 3 g_VS_ L^−1^, 6 g_VS_ L^−1^, 12 g_VS_ L^−1^, and 24 g_VS_ L^−1^, respectively. Methane production per g of VS applied was not significantly different in each experiment, ranging between 294 and 366 mL g^−1^_VS_. However, the increase in feedstock concentration resulted in a prolonged lag phase (Figure 6B). It has been previously shown that the increase in essential oil concentration can negatively impact biomethanization of orange peel waste, inhibiting the methanogenesis [40]. However, the results obtained here demonstrate that anaerobic digestion of CPW under mesophilic conditions can generate high methane yields, comparable to those obtained under thermophilic conditions. Thus, the maximum yield of methane achieved in the present study ranged between 294 and 366 mL g^−1^_VS_, which was three times higher than the yield achieved by Forgacs et al. [17], which reached 102 mL g^−1^_VS_ under thermophilic conditions. A similar effect was reported by Lotito et al. [41], where the use of fresh and stored citrus peel waste achieved methane production that ranged between 333 and 471 mL g^−1^_VS_ under mesophilic conditions. Nevertheless, significant quantities of α-terpineol and p-cymene were detected as final degradation products towards the end of digestion, while d-limonene was no longer present [40,41].

## 3. Materials and Methods 

### 3.1. Citrus Peel Waste

The feedstock applied in the experiments of the present work constituted citrus residues (seeds, pulp, and segment membranes), which were collected from a local citrus processing industry (KEAN Soft Drinks Ltd., Limassol, Cyprus) and maintained at −20 °C until further use. CPW was thawed and ground to solids of less than 2 mm in diameter using a laboratory blender (Waring Commercial, San Antonio, TX, USA). The chemical composition of CPW has been previously determined as follows: 22.00% cellulose, 11.09% hemicellulose, 2.19% lignin, 8.10% glucose, 12.00% fructose, 2.80% sucrose, 25.00% pectin, 6.07% protein, and 3.78% limonene [32].

### 3.2. Pretreatment of Citrus Peel Waste

#### 3.2.1. Extraction of Essential Oils and Pectin

The first step of CPW pretreatment employed distillation for extraction and collection of essential oils as previously described [30], recovering 0.43% *w*/*w* of essential oils. Residues from essential oil distillation were dried at 70 °C for 24 h [42] and dilute acid hydrolysis was performed in an autoclave (SANYO MLS-3781L, Panasonic, Tottori, Japan) at 108 °C, 116 °C, and 125 °C for 10 min and 20 min, using 5% (*w*/*v*) of dry CPW with the addition of 0.5% (*v*/*v*) of sulfuric acid. Following dilute acid hydrolysis, centrifugation and filtration were employed to obtain the supernatant, which was mixed with an equal volume of ethanol (96% *v*/*v*) to precipitate pectin at room temperature for 4 h [32]. Subsequently, the mixture was centrifuged at 3000 rpm for 30 min. The precipitate was washed five times with ethanol (45% *v*/*v*), followed by drying at 50 °C to obtain pectin [43], isolating 23.25% *w*/*w* of the heteropolysaccharide, while the supernatant was applied in distillation at 80 °C three times to recover and recycle ethanol for pectin precipitation.

#### 3.2.2. Enzyme Hydrolysis

Enzyme hydrolysis was applied as additional pretreatment prior to bioethanol fermentations. The procedure was performed with the addition of solid residues from acid treatment in the hydrolyzate obtained following ethanol recovery. The pH of the mixture was adjusted to 4.8 with the use of 1 M NaOH to ensure that process conditions were within the optimal pH range of 4.5–5.0 for the enzymes employed, while cellulases (Chem Cruz, Dallas, TX, USA) and β-glucosidases/pectinases (Oenozym FW, Lamothe-Abiet, Bordeaux, France) were added at 30 IU g^−1^_drm_ and 25 BGL g^−1^_drm_, respectively [30]. Enzyme hydrolysis was performed at 50 °C for 48 h in shake flasks stirred at 100 rpm in a water bath, while upon hydrolysis completion, enzymes were inactivated via heating in an oven at 105 °C for 15 min [44].

### 3.3. Ethanol Fermentations

*P. kudriavzevii* KVMP10 was previously isolated as a thermotolerant ethanologenic yeast within our research group [45], while *K. marxianus* was obtained from the Leibniz Institute DSMZ—German Collection of Microorganisms and Cell Cultures (Braunschweig, Germany). Commercial pressed baker’s yeast was used as a source of *S. cerevisiae*. All strains were maintained at −80 °C in glycerol stock cultures.

*P. kudriavzevii* KVMP10 and *S. cerevisiae* were precultured in liquid media, simulating a Valencia orange peel waste hydrolyzate [4], while the inoculum of *K. marxianus* was prepared using universal media for yeast strains (containing (g L^−1^) yeast extract 3, malt extract 3, peptone 5, and glucose 10) incubated at 30 °C in shake flasks stirred at 100 rpm. Ethanol fermentations were performed in batch experiments using 100 mL flasks with a working volume of 60 mL at 42 °C and 100 rpm. The mixture of reducing sugars obtained from CPW pretreatment (dilute acid hydrolysis only or combined acid and enzyme hydrolysis) was used as a carbon source for the experiments. The feedstock was supplemented with 10 g L^−1^ of yeast extract as a nitrogen source. Moreover, recycling of the remaining liquid stillage following essential oil extraction into the hydrolysis process was tested to enhance the soluble sugar content in the hydrolyzate. All experiments were performed in duplicate, while three samples were analyzed for each replicate, constituting analyses of 6 samples at each time point.

### 3.4. Anaerobic Digestion Experiments 

CPW, DCPW, and BR (remaining solids following acid hydrolysis of CPW) were used as feedstock in anaerobic digestion. Equal quantities of volatile solids (VS, 6 g L^−1^) were employed to produce biogas under mesophilic conditions (37 °C), while anaerobic digestion was performed in batch experiments using 250 mL flasks with a working volume of 150 mL. The nutrient medium was prepared according to the composition used in Angelidaki et al. [46]. Each bottle was supplemented with 6 g of granular sludge withdrawn from a full-scale UASB reactor (Charalambides Christis Ltd., Limassol, Cyprus) used for the treatment of dairy wastewater at pH 6.8–7.3. The content of sludge in lignin and N-compounds, cellulose, and hemicellulose was measured at 0.35%, 0.30%, and 1.40%, respectively. Granular sludge was washed with distilled water and applied as active inoculum (4% *w*/*v*), while each bottle was flushed with 100% CO_2_ gas to ensure anaerobic conditions. Experiments were performed in triplicate and methane accumulation was determined for 121 days, aiming to evaluate the adaptation of anaerobic digestion to CPW, DCPW, and BR. Following 36 days, a refeed of each material (6 g_VS_ L^−1^) was applied in each digestion and a subsequent refeed was also conducted at 78 days. The refeed of each material was performed through withdrawal of 50 mL of digested medium, which was replaced with an equal volume of fresh media and substrate. Nevertheless, any remaining substrate and anaerobic sludge could not be removed, while each bottle was subsequently flushed using 100% CO_2_ gas. Although the ratio of biomass versus substrate might have been potentially different between each batch, digestion conditions as well as the procedure of the refeed was the same for all materials tested, enabling direct comparison between each flask. Furthermore, in order to test the effect of different initial VS contents of CPW, 3, 6, 12, and 24 g L^−1^ were applied in triplicate anaerobic digestion experiments for 92 days, evaluating the effect of essential oils in the process. The sampling procedure included withdrawing 1 mL of biogas with the use of a gas-tight syringe for determination of biogas composition as described in Section 3.5.4., as well as obtaining biomedium samples for the detection of VFAs. During anaerobic digestion, the volume of biogas, methane, carbon dioxide, oxygen, hydrogen, and nitrogen produced was monitored.

### 3.5. Analyses

#### 3.5.1. Ethanol Concentration

Gas Chromatography using a flame ionization detector was employed for the determination of ethanol concentration. A Shimadzu GC-2014 (Shimadzu, Milton Keynes, UK) and a 30 m long Zebron ZB-5 capillary column (Phenomenex, Macclesfield, UK) with a 0.25 mm internal diameter was used. The stationary phase of the column was 5% phenyl- and 95% dimethylpolysiloxane, while the mobile phase applied was nitrogen. Samples obtained during ethanol fermentations were centrifuged for 3 min at 13,000 rpm and the supernatant was filtered through 0.2 μm syringe filters. Ethanol was extracted by vortexing 1 mL of the filtered sample with 2 mL of hexane for 1 min. Approximately 1 μL of the extract was injected and the temperature of the column was kept constant at 40 °C for 3 min. Ethanol concentration was calculated, interpolating from a previously established calibration curve and the coefficient of variation for 3 samples was 1.47% at a concentration level of 4 g L^−1^. Furthermore, the respective LoB, LoD, and LoQ values were calculated at 0, 0.07, and 1.7 g L^−1^, respectively.

#### 3.5.2. Reducing Sugars 

During ethanol fermentations the content of reducing sugars was analyzed by the phenol–sulfuric acid method [47]. The LoB, LoD, and LoQ values were calculated at 0, 1.48, and 11.18 mg L^−1^, respectively.

#### 3.5.3. Total and Volatile Solids 

The total solids (TS) and VS content of granular sludge, CPW, DCPW, and BR was determined according to Standard Methods [48]. The LoB/LoD/LoQ values for the determination of formic acid, acetic acid, propionic acid, butyric acid, and valeric acid concentrations were calculated at 0/0.002/0.3 g L^−1^, 0/0.01/0.3 g L^−1^, 0/0.01/0.2 g L^−1^, 0/0.01/0.2 g L^−1^, and 0/0.003/0.2 g L^−1^, respectively.

#### 3.5.4. Biogas Composition

Biogas composition (H_2_, O_2_, N_2_, CH_4_, and CO_2_) was analyzed using a gas chromatograph (Agilent Technologies, 7820OA, Santa Clara, CA, USA) fitted with a ShinCarbon ST 50/80 (2 m length, 2.2 mm ID) mesh column (Restek Corporation, Bellefonte, PA, USA) and thermal conductivity detector as described by Vardanyan et al. [49]. The LoB/LoD/LoQ values for the determination of hydrogen, oxygen, nitrogen, methane, and carbon dioxide volumes were calculated at 0/0.4/8.4 mL, 0/0.4/3.4 mL, 0/1.2/10.5 mL, 0/0.2/7.0 mL, and 0/0.3/1.1 mL, respectively.

#### 3.5.5. Volatile Fatty Acids (VFAs)

The concentration of VFAs (acetate, formate, butyrate, propionate, and valerate) formed during anaerobic digestion was measured through High Pressure Liquid Chromatography (HPLC). Culture samples were centrifuged at 13,000 rpm for 3 min and filtered using 0.22 mm syringe filters. A Shimadzu LC-20AD liquid chromatograph (Shimadzu, Milton Keynes, UK) equipped with a Shimadzu SPD-20A UV/VIS detector, a Shimadzu SIL-20A HT auto sampler, and a CTO-10AS VP column oven was used. The column was eluted isocratically at a rate of 0.7 mL min^−1^ from an organic analysis column (Rezex ROA-Organic Acid column, Phenomenex, Torrance, CA, USA) with 5 mM H_2_SO_4_ at 55 °C, while the injection volume was 1 μL.

## 4. Conclusions

This paper has demonstrated the potential of a novel zero-waste biorefinery for the production of renewable biofuels from CPW through low environmental impact technologies. It was confirmed that the combination of sequential acid and enzyme hydrolysis for CPW pretreatment enhanced the fermentative production of ethanol. Moreover, the thermotolerant *P. kudriavzevii* KVMP10 was a more efficient ethanol producer at elevated fermentation temperatures as compared to industrial yeasts (*S. cerevisiae* and *K. marxianus*). The use of BR in anaerobic digestion for methane generation resulted in increased production of the biofuel. The study presents important operational parameters reducing the energy requirements of the biorefinery. 

## Figures and Tables

**Table 1:** Consumption of reducing sugars and final titre of ethanol in fermentations of acid hydrolyzates.

Experiment	Reducing Sugars Consumed (g L^−1^)	Final Ethanol Titre (g L^−1^)
***P. kudriavzevii* KVMP10**		
108 °C, 10 min	11.0	4.7
108 °C, 20 min	17.9	5.2
116 °C, 10 min	12.1	5.8
116 °C, 20 min	30.9	2.9
125 °C, 10 min	15.5	5.8
125 °C, 20 min	20.5	6.7
***K. marxianus***		
108 °C, 10 min	9.8	3.6
108 °C, 20 min	3.1	1.6
116 °C, 10 min	17.2	4.6
116 °C, 20 min	10.9	2.3
125 °C, 10 min	7.5	3.8
125 °C, 20 min	18.6	1.8
***S. cerevisiae***		
108 °C, 10 min	6.9	3.5
108 °C, 20 min	5.4	1.8
116 °C, 10 min	8.6	4.2
116 °C, 20 min	6.0	2.7
125 °C, 10 min	8.1	2.2
125 °C, 20 min	5.9	3.0
